# Adaptive PI Controller Based on a Reinforcement Learning Algorithm for Speed Control of a DC Motor

**DOI:** 10.3390/biomimetics8050434

**Published:** 2023-09-19

**Authors:** Ulbio Alejandro-Sanjines, Anthony Maisincho-Jivaja, Victor Asanza, Leandro L. Lorente-Leyva, Diego H. Peluffo-Ordóñez

**Affiliations:** 1 Escuela Superior Politécnica del Litoral, Guayaquil 090903, Ecuador; ualejand@espol.edu.ec (U.A.-S.); amaisinc@espol.edu.ec (A.M.-J.); 2 SDAS Research Group, Ben Guerir 43150, Morocco; victor.asanza@sdas-group.com (V.A.); leandro.lorente@sdas-group.com (L.L.L.-L.); 3 Faculty of Law, Administrative and Social Sciences, Universidad UTE, Quito 170147, Ecuador; 4 College of Computing, Mohammed VI Polytechnic University, Ben Guerir 47963, Morocco; 5 Faculty of Engineering, Corporación Universitaria Autónoma de Nariño, Pasto 520001, Colombia

**Keywords:** reinforcement learning, artificial intelligence, adaptive PI, DDPG TD3, neural network

## Abstract

Automated industrial processes require a controller to obtain an output signal similar to the reference indicated by the user. There are controllers such as PIDs, which are efficient if the system does not change its initial conditions. However, if this is not the case, the controller must be retuned, affecting production times. In this work, an adaptive PID controller is developed for a DC motor speed plant using an artificial intelligence algorithm based on reinforcement learning. This algorithm uses an actor–critic agent, where its objective is to optimize the actor’s policy and train a critic for rewards. This will generate the appropriate gains without the need to know the system. The Deep Deterministic Policy Gradient with Twin Delayed (DDPG TD3) was used, with a network composed of 300 neurons for the agent’s learning. Finally, the performance of the obtained controller is compared with a classical control one using a cost function.

## 1. Introduction

In an industrial process, the control variables must be considered to ensure that the final product meets high quality standards. A classical control technique is to apply a Proportional–Integral–Derivative (PID) controller, and it is estimated that around 95% of closed-loop systems adopt this type of family, mainly PI [1]. Once the three terms are turned, it is important that the initial conditions of the industrial plant remain uncharged, as they would become inefficient in the face of new dynamic characteristics in the output. It is possible to determine the system’s dynamics through mathematical methods, where by considering all the equations that affect the plant a transfer function in the frequency domain *S* can be obtained, and with the use of MATLAB software, the controller constants can be obtained. Another alternative is to perform an open-loop test on the system, acquiring its data and preprocessing them so that with the help of the System Identification or Sisotool by MATLAB, a mathematical representation can be found and the PID controller can be obtained. The first described process is impractical, since it is possible to make mistakes due to all the conditions that are taken to model the plant, in addition to the existence of a complex system to model. The second option requires a test where the plant must undergo high levels of production, which is why it is carried out with the operators’ consent and at established times to consider minimal production losses due to the tests performed [2].

In the field of control engineering, controller design is fundamental to ensuring the optimal and secure operation of dynamic systems [3,4,5]. The ability to influence the behavior of systems and processes through the manipulation of inputs is essential for achieving specific objectives. However, this task is not without challenges and complexities. Therefore, it is necessary to thoroughly explore the process, key concerns, and the fundamental relevance of concepts such as stability and robustness [6,7,8]. Stability refers to a system’s ability to remain bounded and converge towards a desired state over time, avoiding undesirable oscillations or divergence, while robustness relates to a controller’s ability to maintain satisfactory performance despite uncertainties, operational changes, or external disturbances [9,10]. In this context, the necessity to prevent severe consequences due to instability and to adapt to differences between theory and reality to maintain predictable performance is framed.

The process of controller design starts with the formulation of mathematical models representing the systems to be controlled and evolves through the selection of control strategies and the implementation of algorithms [11]. Aimed at regulating the system’s outputs, controller design faces challenges due to the diversity of systems, their often nonlinear dynamic behavior, and the necessity to address multiple objectives simultaneously.

The PID controller, renowned for its responsiveness and stability, has been widely used in industrial control systems [12,13,14,15]. However, its performance can be hindered by the need for manual adjustment of parameters for each specific application. Alternatively, artificial intelligence techniques, particularly reinforcement learning algorithms, provide the option to automatically and continuously adjust controllers based on real-time feedback obtained from the system [16,17,18].

For the reasons described above, a PID controller will be used whose tuning is based on reinforcement learning. This controller will adapt to the environment and its changes thanks to its artificial intelligence, which is constantly learning. Nowadays, due to the complexity of the term, there are many definitions of artificial intelligence (AI). A simplification of the definition would be “Computer ability to perform activities that normally require human intelligence”, as explained by Lasse Rouhianen [19]. Machine learning is a subfield of AI that provides computers with the ability to learn without being explicitly programmed [20]. The *machine learning* (ML) process has two stages, one of which is training, which involves the system trying to learn behaviors or patterns that fit the data from the training set. The second stage is prediction, which refers to the output of an algorithm after it has been trained with a set of historical data and applied to new data to predict the probability of a particular outcome [21]. Machine learning uses algorithms based on mathematics and statistics to discover patterns in data [22,23,24,25,26,27].

**Figure 1:**
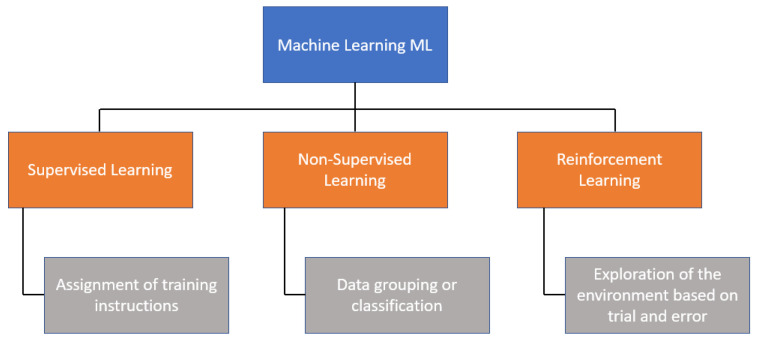
Classification of machine learning.

There are three main categories of ML algorithms, as shown in Figure 1. One of them is *supervised learning*, which uses a set of input data to obtain an expected outcome from the user; then, there is *unsupervised learning*, which involves a set of input data, but no specific task or objective is specified for the algorithm. Finally, there is *reinforcement learning* (RL), which is an algorithm that learns by observing and interacting with the environment, where its decisions are rewarded if they are correct; otherwise, it receives penalties.

**Figure 2:**
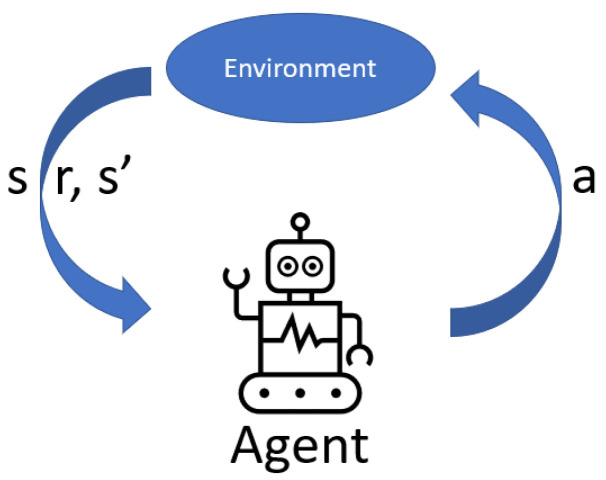
Reinforcement learning.

Reinforcement learning is centered on acquiring knowledge about which actions to undertake and how to assign these actions to various situations, with the aim of maximizing a numerical reward [28,29,30,31,32]. Scenarios are presented where the actions taken can impact not only immediate rewards but also future states and rewards, leading to a methodology based on trial and error, alongside deferred rewards. In this approach, there is no authoritative figure dictating actions to the system; instead, a simple reward signal exists, and feedback from the environment is not immediate but delayed. The system’s decisions affect the information that will be received in subsequent stages [33]. Within this methodology, two main components are highlighted: the agent and the environment. The objective is to ensure that the agent continuously interacts with the environment with the goal of achieving a specific objective, as depicted in Figure 2.

There are other elements that intervene in reinforcement learning: the policy, value function, and reward. Reinforcement learning has two main approaches: One is the passive approach, where algorithms have a fixed policy, meaning it does not change. The other is the active approach, also known as Q-Learning, which calculates the policy by testing actions; this involves slow learning, because it requires exploration [34].

The purpose of this paper is to adjust a PI controller to control the speed of a DC motor. Two PI controllers are obtained, and an NARMA-L2 controller is compared against classic control and reinforcement learning to evaluate the metrics of the step responses.

The first one uses classical control techniques where it is necessary to know the transfer function and then use the Sisotool, which is a MATLAB tool, and the PI controller is adjusted based on the user’s requirements and the system.

The second controller is generated by the artificial intelligence of our agent. The third NARMA-L2 controller is configured using Simulink and is generated by the neural networks of the controller based on its plant identification. The plant was modeled in a different Simulink environment. It is important to note that if the system changes its material conditions for any reason, or even during preventive maintenance, re-engineering processes must be carried out to assess the new transfer function (mathematical modeling) and design its PI controller. When applying reinforcement learning techniques, these efforts are meticulous, because the system learns based on its conditions and adjusts automatically without halting the process.

The performance of both controllers is estimated and compared based on the cost function, which indicates how much effort the controller has made to reach its reference signal adjusted by the user. The times it takes the agent to learn from the system are shown in order to evaluate them with the computational resources that were used in this work. Finally, the graphs demonstrate how assertive it is to apply modern control vs classical control to nonlinear systems, as well as visualize all the time transitions that arose when finding the correct gains of the trained agent.

The remainder of this paper is structured as follows: Section 2 presents some related works on DC motors, controllers, and the application of reinforcement learning algorithms. Section 3 describes the system design, including modeling, parameters, data acquisition, and speed control for a DC motor. Section 4 presents the experimental results and discusses their implications. Finally, Section 5 provides the conclusions of this study and outlines future work.

## 2. Related Works

In the industrial sector, the use of direct current (DC) motors is of crucial importance due to their wide range of applications [35]. These motors are highly valued for their efficiency and versatility. However, to ensure optimal performance, it is essential to have precise control over the speed of these motors. In this regard, PID controllers are widely recognized and have proven to be an effective and reliable solution [36,37,38]. It is important to consider that the proper implementation and configuration of PID controllers are key elements to achieve optimal performance.

A series of investigations have been conducted focusing on the usefulness of PID controllers in industrial environments, such as regulating the speed in automated transportation systems [39]. This approach has even extended to specific areas like assembly lines [40,41,42]. An example of such an application is found in the automotive industry, where Farag in 2020 [43] proposed a PID controller to enhance the maneuverability of autonomous vehicles on roadways. PID controllers have also been considered to adjust the speed of conveyor belts, ensuring an appropriate and coordinated flow of components in the assembly process [44,45]. Other works [46] adopt the PID algorithm for controlling the motion of a robot, specifically to regulate the speed of two DC motors and achieve precise control of a mobile platform’s velocity. Furthermore, according to Carlucho et al. 2020 [47], the simplicity of the PID controller means it remains widely used in industrial applications and in the field of robotics.

However, it is crucial to acknowledge that the successful implementation of PID controllers and, more generally, controller design, face challenges and concerns inherent to the field of control engineering [48,49]. A system’s stability and ability to cope with disturbances and changes in operating conditions are critical considerations [50,51]. Additionally, achieving a balance between controller performance and robustness becomes imperative. In fact, a controller with outstanding performance but limited ability to handle disturbances can lead to unsatisfactory outcomes in challenging industrial environments [52,53,54,55]. Consequently, the design and tuning of controller parameters must be approached carefully to ensure both optimal performance and reliable stability [56,57].

The application of direct current motors in the industrial realm underscores the critical importance of precise speed control. The adoption of PID controllers in particular reinforces this notion by providing a dependable solution. However, the success of implementation lies in appropriate design and tuning, considering concerns related to stability, robustness, and the balance between performance and reliability in industrial contexts [58,59,60].

In the field of DC motor control, there has been a constant quest to improve the precision and efficiency of control systems. In this context, the use of reinforcement learning algorithms has emerged as a promising technique to adapt controllers in real-time and optimize their performance [61,62,63].

Several studies have used reinforcement learning algorithms to improve the performance of PID controllers in the efficient and optimal control of DC motors [64,65,66,67,68,69]. In this regard, in [64], the use of deep reinforcement learning is proposed to enhance the classical PID controller. They present a new adaptive controller whose parameters are adjusted in real-time according to the constantly changing state. Following this line, in [65], the same algorithm is employed to modulate the gain parameters of a fuzzy PID controller, successfully improving the accuracy of the fuzzy control and minimizing the error for precise control performance. In another approach, the authors of the article [66] propose an adaptive controller based on deep reinforcement learning to improve the control of a DC motor powered by a DC–DC Buck converter. Alternatively, in [61], the researchers develop a hybrid reinforcement learning approach that combines the Zeigler–Nichols method with fuzzy Q-Learning for real-time tuning of PID parameters in the speed control of a DC motor. In further research [67], a theoretical demonstration of reinforcement learning for the dynamics of the PI controller is conducted to achieve the optimal speed control of DC motors using the Twin Delay algorithm of deep deterministic policy gradient, yielding very promising results.

In this paper, we explore a novel perspective on speed control in DC motors. By utilizing an adaptive PI controller based on a reinforcement learning algorithm, we provide the capability for automatic and continuous adjustment of controller parameters. This results in more precise and efficient motor control. The results obtained in this study lay the foundation for future research in the field of adaptive control and optimization of motor control systems.

## 3. System Design

This section describes the mathematical modeling of the DC motor, experimental measurement of the DC motor parameters, data acquisition from the DC motor, and design of the DC motor speed control based on a reinforcement learning algorithm.

### 3.1. DC Motor Modeling

Mathematical modeling involves representing the system (DC motor) through equations, which can be differential depending on the elements that constitute them. For this, a high degree of physical understanding, as well as of disturbances, is required, which is why it is considered a rigorous process that requires several conditions to finally obtain a transfer function in the *S* frequency domain through Laplace conversion.

**Figure 3:**
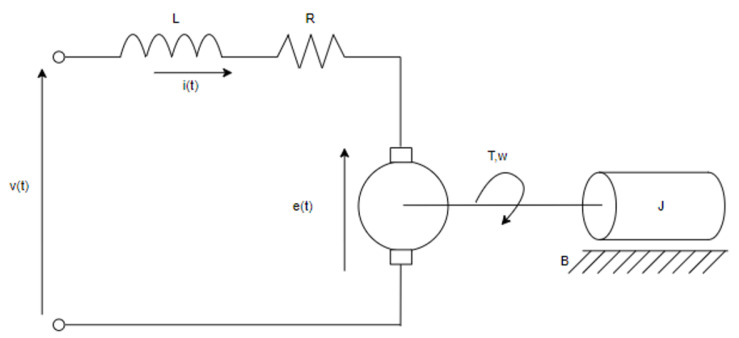
Electric model of the DC motor.

The transfer function allows for analyzing the dynamic and static behavior of the system, enabling the extraction of control system characteristics such as overshoot percentage, steady-state time, steady-state output, peak time, and tau, among others. The system’s response is pivotal, as the transfer function establishes a relationship between input and output, enabling project sizing prior to execution and, in the case of the motor, determining appropriate protections or controls. Additionally, through the use of the transfer function and control theory, it was possible to size the PID controllers, which are adjusted based on the type of system previously studied. Nonetheless, the current conditions and variables of the DC motor are also presented. For reference regarding the electrical system’s acquisition of its transfer function, Figure 3 is provided.

It is important to consider the units of measurement based on their symbols (see Table 1). This allows for adapting the formulas and simplifying their derivation. Given the system constants, the equations that represent the system are obtained, starting with the closed loop in the armature:

(1) $$v\left(t\right)=Ri\left(t\right)+L\frac{di\left(t\right)}{dt}+e_{a}\left(t\right)$$

(2) $$L\frac{di\left(t\right)}{dt}=v\left(t\right)-e_{a}\left(t\right)-Ri\left(t\right)$$

The equations that describe the mechanical section are

(3) $$T_{m}\left(t\right)=J\frac{d\omega \left(t\right)}{dt}+B\omega \left(t\right)$$

(4) $$J\frac{d\omega \left(t\right)}{dt}=T_{m}\left(t\right)-B\omega \left(t\right)$$

The counter-electromotive force is defined by a constant, in addition to being directly proportional to the angular speed with which the motor rotates, therefore,

(5) $$e_{a}\left(t\right)=f\left\{\omega \left(t\right)\right\}=k_{a}\omega \left(t\right)$$

The mechanical–electromechanical relationship is defined as a constant multiplied with the armature current:

(6) $$T_{m}\left(t\right)=f\left\{i\left(t\right)\right\}=k_{m}i\left(t\right)$$

The four aforementioned equations define the behavior of the system. Now, the Laplace transform is applied, obtaining the following:

(7) $$LsI\left(s\right)=V\left(s\right)-RI\left(s\right)-E_{a}\left(s\right)$$

(8) $$Js\omega \left(s\right)=T_{m}\left(s\right)-B\omega \left(s\right)$$

(9) $$E_{a}\left(s\right)=k_{a}\omega \left(s\right)$$

(10) $$T_{m}\left(s\right)=k_{m}I\left(s\right)$$

Solving the current in the Equation (10):

(11) $$I\left(s\right)=\frac{T_{m}\left(s\right)}{k_{m}}$$

From Equations (8)–(10), the angular speed–voltage transfer function will be obtained; in this way, the voltage applied to the armature can be varied using the PWM technique to control the speed of the DC motor.

(12) $$Js\omega \left(s\right)=T_{m}\left(s\right)-B\omega \left(s\right)$$

(13) $$Js\omega \left(s\right)=k_{m}I\left(s\right)-B\omega \left(s\right)$$

Solving the current:

(14) $$I\left(s\right)=\frac{Js+B}{k_{m}}\omega \left(s\right)$$

Substitute Equations (14) and (9) in (7):

(15) $$Ls\left(\frac{Js+B}{k_{m}}\omega \left(s\right)\right)=V\left(s\right)-R\left(\frac{Js+B}{k_{m}}\omega \left(s\right)\right)-k_{a}\omega \left(s\right)$$

Finally, we note that the equation depends on the voltage applied to the armature and the angular velocity; so, we only need to solve for it to obtain the transfer function, which will allow us to find the PID constants using classical and modern control techniques.

(16) $$\frac{\omega \left(s\right)}{V\left(s\right)}=\frac{k_{m}}{\left(LJ\right)s^{2}+\left(LB+RJ\right)s+\left(RB+k_{a}k_{m}\right)}$$

### 3.2. Experimental Measurement of DC Motor Parameters

**Figure 4:**
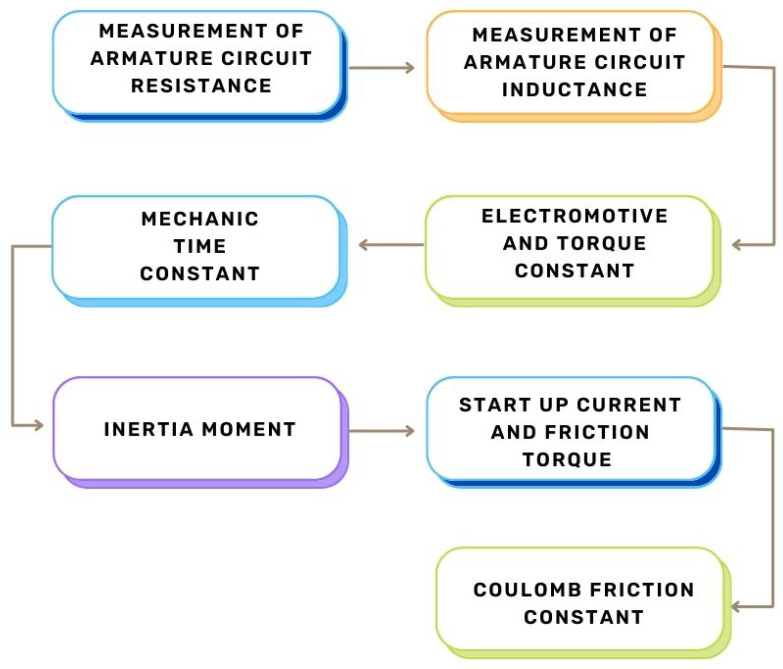
Processes used to obtain the experimental parameters of the DC motor.

The goal of obtaining experimental parameters for the DC motor is to accurately derive a transfer function that represents the dynamics of a real plant. While an artificial intelligence algorithm is not required to understand the system, for this work, we simulate the plant using a transfer function to derive two PID controllers: one using modern control and the other using classical control. The process is detailed in Figure 4.

**Figure 5:**
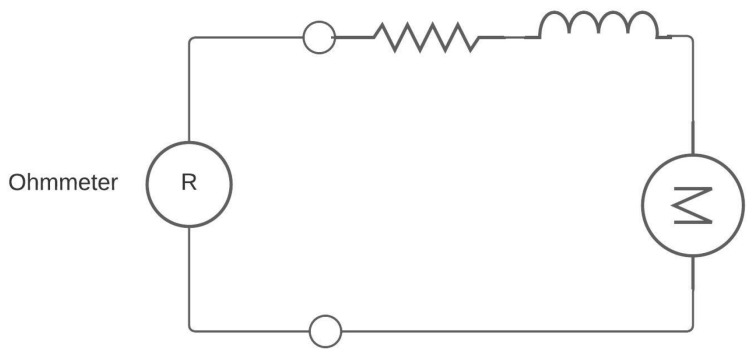
Multimeter connection with DC motor.

The DC motor used has the following characteristics (Table 2):

- Armature resistance: To measure the armature winding resistance, we use a multimeter set to Ohms and then establish the connection, as shown in Figure 5.
- Armature inductance: The armature inductance, like the armature resistance, is measured in the windings of the DC motor using an instrument called an LCR meter, which is used for measuring inductances.
- Electromotive force and torque constant: To obtain the electromotive force and torque constant, we need to use the mathematical modeling of the DC motor and equations derived from Kirchhoff’s voltage law.

(17) $$k_{a}=\frac{E_{a}}{\omega \left(t\right)}$$

(18) $$V=iR+E_{a}$$

(19) $$k_{a}=\frac{V-iR}{\omega \left(t\right)}$$

Equation (19) indicates that we must measure the voltage applied to the armature, current, and speed in radians per second. For this purpose, the following system was proposed.

**Figure 6:**
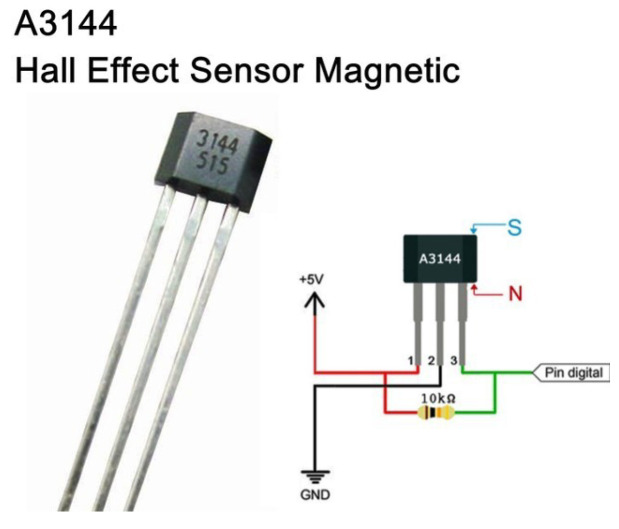
A3144 Hall effect sensor.

**Figure 7:**
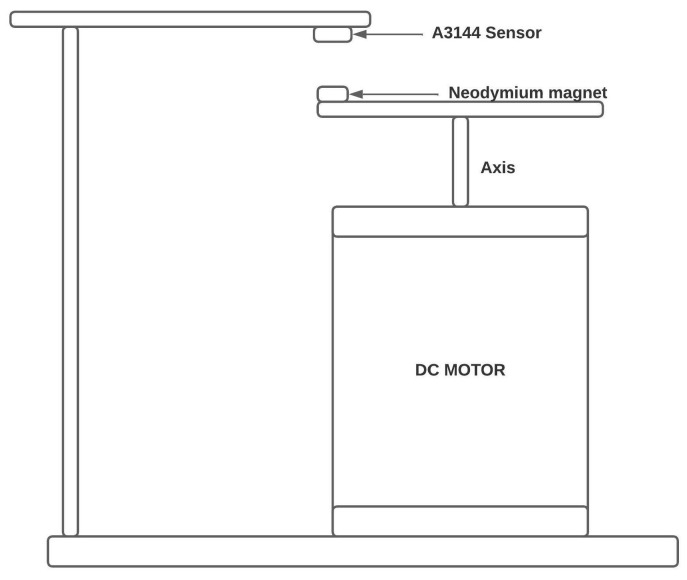
RPM measuring system.

The A3144 sensor, a Hall effect sensor, was used (see Figure 6). Its main function is to detect magnetic fields, and it has the advantage of retaining states. In other words, it changes from a Boolean value of 0 to 1 based on the current state, enabling accurate detection of rising pulses upon detecting magnetic fields. Reference to its assembly can be found in Figure 7.

**Figure 8:**
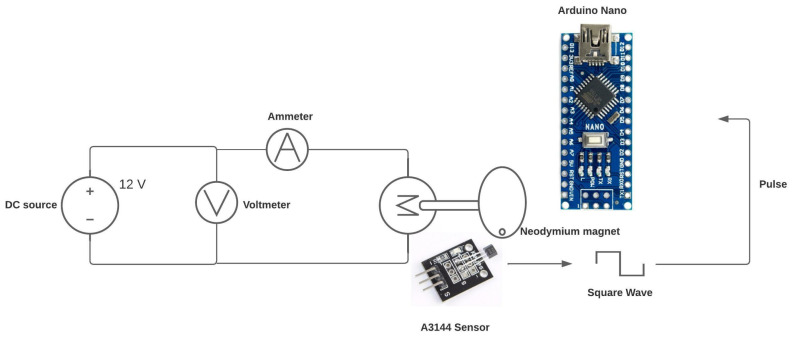
Sensor measurement.

The following schematic diagram (see Figure 8) explains how RPM will be obtained using the Hall effect sensor and a microcontroller. In this case, the Arduino Nano development board is used, which has the Atmega328PB microcontroller.

The voltage and current values for this test were obtained using the multimeter. However, for the speed measurement, a code was used that allowed obtaining this variable by counting the time between pulses that arrive at pin 2 of the Arduino Nano.

It is important to use this technique, because if we count the number of turns our motor has made in a certain amount of time, we would obtain inaccurate readings of the speed, since only complete turns are taken into account.

This algorithm detects a pulse and starts counting time using an Arduino function until it detects another pulse. This way, a period is obtained, which can be converted into frequency and then into revolutions per minute. The following flowchart explains the programming algorithm used for this task (Algorithm 1).

**Algorithm 1** RPM measurement Start Calibrate number of magnets and samples Start serial communication **if** Rising edge pin 2 **then**     Start function “micros()”             ▹ The function starts a timer     **if** First rising edge? **then**         lastTimeWeMeasured = micros()         **if** Second rising edge? **then**            PeriodBetweenPulse = micros()-lastTimeWeMeasured            Frequency and rpm calculation         **else**            lastTimeWeMeasured = micros()         **end if**     **else**         Start function “micros()”     **end if** **else**     Wait for rising edge **end if**

**Figure 9:**
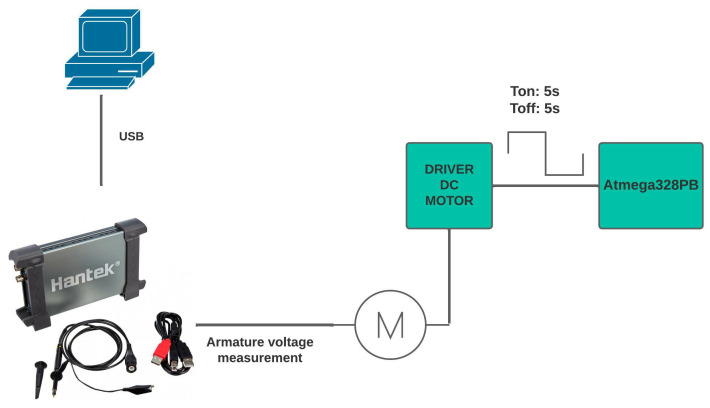
Measurement of the mechanical time constant.

- Mechanic time constant: Now, we proceed to determine the mechanical time constant, which represents 63.2% of the system stabilization time. To perform this test, a code was created to keep the motor on for 5 s and then turn it off for 5 s. This aims to observe the system dynamics and measure the stabilization time. The equipment used is the HANTEK6022BE PC-OSCILLOSCOPE, which shows us the behavior of the voltage curve generated during the transient, taking advantage of the fact that the speed is directly related to the voltage applied to the armature; its acquisition is detailed in the Figure 9.
- Inertia moment: With all the obtained parameters, the following equation can be used to obtain this constant:
  (20) $$J=\frac{T_{m}\cdot k_{a}\cdot k_{m}}{R}$$
- Start up current and friction torque: It is also possible to determine the starting current and friction torque by using a variable DC voltage power supply, gradually applying voltage to the DC motor armature and determining the necessary current for the rotor to begin turning. With the starting current, the torque constant caused by friction on the shaft can be determined. However, this test could not be performed, because the instrument needed to regulate the voltage was not available.
  (21) $$T_{f}=k_{m}\cdot i_{start}$$
- Coulomb friction constant: Finally, the Coulomb friction constant is determined, which can be obtained quickly by applying the following equation.
  (22) $$B=\frac{T_{m}}{\omega \left(t\right)}$$
  The speed used is the one reached in steady state, and the mechanical time constant found in the previous experiment can be used.

### 3.3. Data Acquisition between Simulink and Arduino

**Figure 10:**
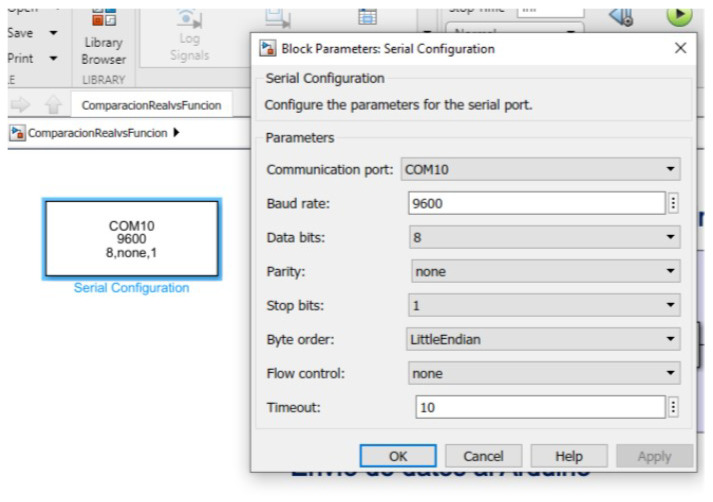
Simulink model of the control system environment.

Serial communication was established between Arduino and Simulink using the “Instrument Control Toolbox”. The objective was to send the RPM values from Arduino and visualize them in Simulink. The algorithm that allows obtaining the RPM from the time between pulses detected by the Hall effect sensor was executed in Arduino. The Simulink configuration is shown in Figure 10.

### 3.4. PI Controller Using Sisotool Toolbox Matlab

Once the transfer function of the system is obtained and located in the MATLAB code, the controller is adjusted using the command Sisotool (G), where G represents the transfer function of the system (Equation (16)). This command is executed, and through its interface, the controller gains are adjusted. Initially, an integrator must be added to the controller so that the output signal of the system approaches zero (steady state), and then the necessary zeros are gradually adjusted to the control system.

In the context of control systems, robustness and stability are two key concepts that refer to a system’s ability to maintain proper operation in the face of disturbances, variations in parameters, or altered conditions. As initial conditions, in this work, it is defined that the settling time should be less than 4 s and have an approximately zero steady-state error (final reference point).

In this work, the aim is to perform a comparison between the controller tuning obtained using the conventional method and the one generated through the reinforcement learning technique. In the Sisotool tool, the controller’s conditions depend on the user’s criteria for adjustment. However, the intention is to evaluate the tuning of the controller generated by neural networks based on the conditions manipulated by the user.

The controller defined by the Sisotool tool is as follows:

(23) $$Controller=\frac{0.00311\cdot (s+9.614)}{s}$$

If any of the conditions change in the control system, all engineering processes must be carried out again to obtain an appropriate transfer function and adjust the PI controller.

### 3.5. Speed Control in a DC Motor Based on a Reinforcement Learning Algorithm

In this section, we discuss how to tune a PI controller using the twin delayed deep deterministic policy gradient (TD3) reinforcement learning algorithm. The performance of the tuned controller is compared with that of a controller tuned using MATLAB’s Sisotool application.

In relatively simple control tasks with a small number of tunable parameters, model-based techniques such as Sisotool, PID Tuner, etc., can achieve good results with a faster tuning process than model-free RL methods. However, RL methods may be more suitable for highly nonlinear systems or for tuning adaptive controllers, which change according to modifications in the system, such as increased load on the shaft.

To facilitate the comparison of controllers, both tuning methods use a Gaussian quadratic linear objective function, where a reinforcement learning agent is used to compute the gains of a PI controller.

#### 3.5.1. Characteristics of MATLAB Software Processing

In this journal, MATLAB version 9.11 R2021b was used, which includes the following toolboxes:

- Simulink version 10.4 (R2021b).
- Deep Learning version 14.3 (R2021b).
- Reinforcement Learning version 2.1 (R2021b).

Furthermore, MATLAB provides the capability to tune a PID controller using control techniques such as Sisotool, PID Tuner, Control System Tuner, etc.

#### 3.5.2. Model of the Environment

**Figure 11:**
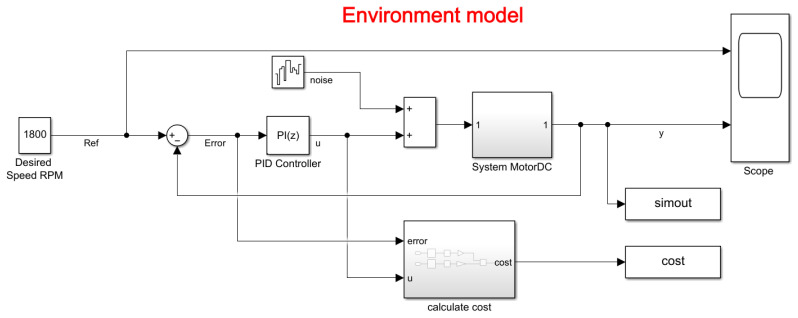
Simulink model of the control system environment.

The environment model is of a DC motor (as shown in the Figure 11). The objective of this control system is to maintain the motor speed to match a reference value. The mathematical model of the aforementioned system allowed it to be modeled in Simulink and thus obtain the input voltage and the output in RPM speed.

In the model, it can be observed that it contains the PID block, which adjusts the PI constants defined by the user or the RL algorithm. In addition, it contains noise in the input signal that is added to the action signal of the controller, so that with or without disturbances, the system has a stable behavior, which is defined with a variance of

(24) $$E(n^{2}\left(t\right))=1$$

The white noise is defined as a stochastic process, which means it has no statistical correlation with its behavior. For optimal control in this system, the LQG criterion is applied, which allows the analysis of costs regarding how much effort the control signal takes to reach the steady-state point set by the user in the dynamic operation of the controlled plant. This is defined below:

(25) $$J=\int_{T_{0}}^{T_{f}}e\left(t\right)dt$$

where:

- *J*: Cost function.
- *E*(*t*): Error as time elapses during simulation.

The Equation (25) represents the cost function in the system, and the error corresponds to the Equation (26).

(26) e(t)=r−v(t)

where:

- *r*: Desired speed reference.
- *v*(*t*): Variable speed over time (system speed)

The cost equation is represented by squared indices:

(27) $$J=\int_{T_{0}}^{T_{f}}e{\left(t\right)}^{2}dt$$

As this function not only depends on the system output but also on the input, it is governed by the control action signal, yielding the following equation:

(28) $$J=\int_{T_{0}}^{T_{f}}\left(e{\left(t\right)}^{2}-pu{\left(t\right)}^{2}\right)dt$$

where:

- *p*: Input weighting.
- *u*(*t*): Control time-varying action signal.

The input weighting allows to ensure that when there are high control signals and the error is minimal, it does not negatively affect the learning and cost analysis of the system. Therefore, to maintain the motor speed while minimizing the control effort u, the controllers in this project used the following LQG criterion:

(29) $$J=\lim_{T\to \infty }E\left(\frac{1}{T}\int_{T_{0}}^{T_{f}}\left({\left(r-v\right)}^{2}\left(t\right)+0.01u^{2}\left(t\right)\right)dt\right)$$

where:

- A weighting of 0.01 was used for the control signal actions.
- *T*: Simulation time in the cost function.

The time used for sampling is 0.1 s, and the simulation time corresponds to 10 s.

#### 3.5.3. Tuning PI Controller Using Sisotool Toolbox in MATLAB

Once the transfer function of the system is obtained and placed in the MATLAB code, the controller is tuned using the Sisotool command in the MATLAB Sisotool Toolbox. By executing the command Sisotool (G), where G represents the transfer function, the controller gains are adjusted through the Sisotool interface. In the first instance, an integrator is added to the controller to ensure that the output signal of the system is very close to zero. A controller is tuned in this toolbox, because a comparative analysis is performed between classical and modern control.

In the code, the transfer function of the controller is written to obtain its gains.

(30) $$Controller=K_{p}+K_{i}\frac{1}{s}$$

where:

- *K_p_*: Controller Proportional Gain.
- *K_i_*: Controller Integral Gain.
- *s*: Frequency domain.

Obtaining the following results:

(31) $$K_{p}=0.00311$$

(32) $$K_{i}=0.0299$$

#### 3.5.4. Creating the Environment for Training the RL Agent

To define the training model for the RL agent, the *Simulink* parameter in the PID controller for the DC motor is modified with the following steps:

1.-Delete the PID Controller.

2.-Insert an RL Agent block.

3.- Create the observation vector (33), where *e* represents the error of the difference between the reference and the speed of the motor. Connect the observer signal to the RL Agent block.

4.-Define the reward function (34) for the RL agent as the negative of the LQG criterion cost, represented in the equation. The RL agent maximizes this reward, thus minimizing the LQG cost.

(33) $${\left[\begin{array}{cc} \int edt & e \end{array}\right]}^{2}$$

(34) $$Rewards=-\left({\left(ref-v\right)}^{2}\left(t\right)+0.01u^{2}\left(t\right)\right)$$

**Figure 12:**
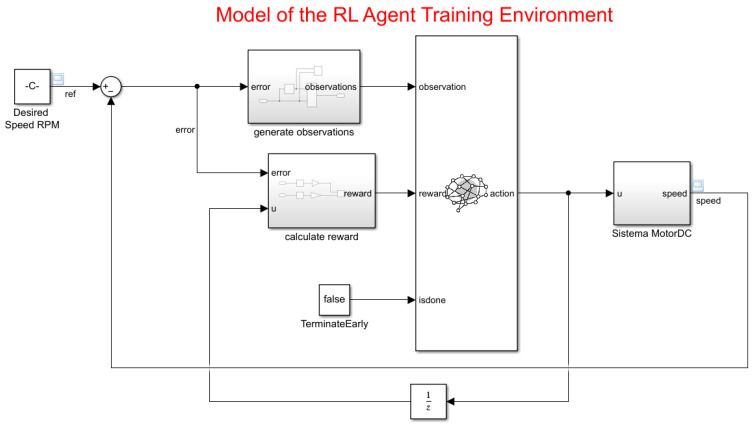
Model of the RL agent training environment.

The results of the changes are presented in Figure 12.

The environment interface was created using the *localCreatePIDEnv* function defined in the MATLAB script, thus extracting the observation and action dimensions from it. The reproducibility random generator is of type *Twister*, which is essential for varying the reference value in model learning.

#### 3.5.5. Creation of the TD3 Agent

Given the observations, a TD3 agent decides which action to take using an actor representation. To create the actor, first, a deep neural network is created with the observation as input and the action as output. The configuration in Simulink is shown in Figure 12.

A PI controller can be modeled as a neural network with a fully connected layer with observations of error and integral of error.

(35) $$u=\left[\begin{array}{cc} \int edt & e \end{array}\right]*{\left[\begin{array}{cc} K_{i} & K_{p} \end{array}\right]}^{T}$$

where:

- *u*: Output of the actor neural network.
- *K_i_* *y* *K_p_*: Absolute values of the weights in the neural network.
- *e*: Error of the difference between the reference and the actual motor speed.

The gradual descent optimization can lead the weights to negative values. To avoid negative weights, the normal *fullyConnectedLayer* was replaced by a *fullyConnectedPILayer*. This layer ensures that the weights are positive, and it is implemented in the function (36), and it was defined as *fullyConnectedPILayer.m*.

(36) Y=abs(WEIGHTS)∗X

A TD3 agent approximates the long-term reward given the observations and actions using two value function representations. To create the critics, first, a deep neural network is created with two inputs, the observation and the action, and one output.

**Figure 13:**
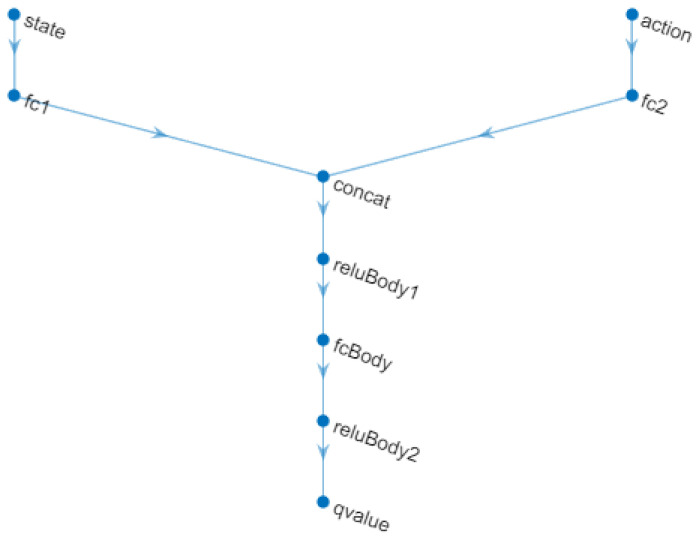
Critic Neural Network.

To create the critics, the *localCreateCriticNetwork* function defined at the end of the MATLAB script was used. The same network structure was used for both critic representations. This neural network is shown in Figure 13.

Two critics are used, because each one can learn about the gains of both the proportional and integral constants individually, as they are very different values in the adjustment of their parameters and must have their own learning in the system.

In addition, the agent must be configured for this project as follows:

- Agent configuration to use the control sample time *Ts*.
- Adjust the mini-batch size to 128 experience samples.
- Set experience buffer length to $1\times 10^{6}$.
- Set the exploration model and target policy smoothing model to use Gaussian noise with variance 0.1.

It is possible to use the functions proposed by MATLAB, such as *rlTD3AgentOptions*, which configures the TD3 agent options, where as *rlTD3Agent* allows one to create the TD3 agent using actor rendering, critic rendering, and the agent options proposed above.

#### 3.5.6. Options for Agent Training

To train the agent, the training options are first specified:

- Each training is run for a maximum of 1000 episodes, with each episode lasting a maximum of 100 time steps.
- Show the training progress in the Episode Manager (set the Plots option) and disable the command line display (set the Verbose option).
- Training is stopped when the agent receives an average cumulative reward greater than −355 in 100 consecutive episodes. At this point, the agent can control the speed of the DC motor.

All these options allow to obtain a better analysis plot with respect to the training times, which contain signals of both the average and the target Q, the latter indicating the training experiences that in each episode try to drive a gain of the PI controller. The training was conducted on a computer with the technical specifications indicated in Table 3.

### 3.6. Control of a DC Motor Using the NARMA-L2 Controller

**Figure 14:**
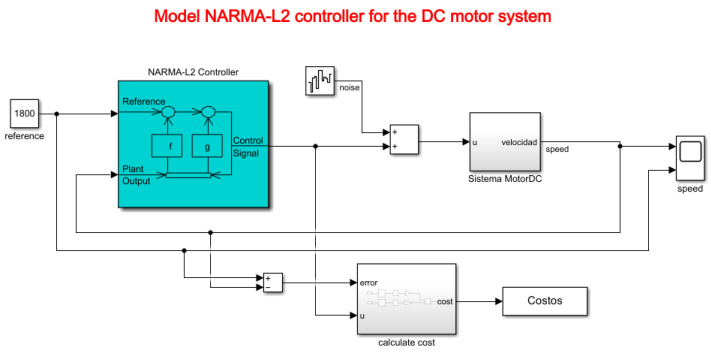
Model of the NARMA-L2 controller for the DC motor system.

The NARMA-L2 controller (Nonlinear AutoRegressive Moving Average with exogenous inputs—Second order) is a type of controller used in nonlinear control systems. This controller is based on a nonlinear mathematical model to estimate the response of a dynamic system and generate appropriate control signals to maintain the system in a desired state. The system used in this article is a DC motor, for which all constants were modeled based on experimentation in Section 3.2. Figure 14 depicts the Simulink model proposed for controller testing.

The term “NARMA” refers to “Nonlinear AutoRegressive Moving Average”, which indicates that the controller incorporates autoregressive and moving average elements but in a nonlinear form. “L2” refers to its second-order nature, in accordance with Equation (16), indicating that it considers the two most recent samples of the input signal to calculate the control signal.

Using the NARMA-L2 model, the controller is defined by Equation (37).

(37) $$u\left(k+1\right)=\frac{y_{r}\left(k+d\right)-f\left[y(k),...,y(k-n+1),u(k),...,u(k-n+1)\right]}{g[y(k),...,y(k-n+1),u(k),...,u(k-n+1)]}$$

Regarding Equation (37), it is indicated that there are two neural network approximations in the functions *f*() and *g*(), which are associated and allow obtaining a control signal *u* that is fed back to the controller based on the iterations specified in the model. Finally, *y* represents the reference signal of the plant, in this case, the motor speed in RPM. Its representation is shown in the controller model in Figure 14.

For educational purposes, a white noise component is added to the system to analyze how the controller behaves in the presence of disturbances that may occur in the control plant (DC motor).

## 4. Results and Discussion

This section presents: (a) the result of the process in the training of the agent, (b) the result of the process in the training of the NARMA-L2 controller, and (c) the comparison of the efficiencies in the applied models.

### 4.1. Agent Training Process

The agent is trained using the *train* function as part of the *Reinforcement Learning Toolbox* library. Training this agent is a computationally intensive process that takes several minutes (even hours) to complete. To save time when running this project, it is possible to use a pretrained agent by loading it into the MATLAB workspace and thus obtain the weights from the PI controller.

It is important to specify that the training was carried out on a laptop with the following computational resources (Table 3):

In the reinforcement learning interface, three types of graphs are highlighted: Episode reward, Average reward, and Q0 Episode. The light blue curves are the episodes of the reward, which determine if the actions taken by the environment are the most efficient for them to be acted on in the system and thus have the desired speed reference, giving small evaluations in case it reaches its objective. If it does not reach its objective, it receives punishments, which indicates to the RL agent that its actions are not correct and that it must carry out different operations. The behavior of the rewards is evaluated by means of the average through the number of episodes, which stands out in blue.

**Figure 15:**
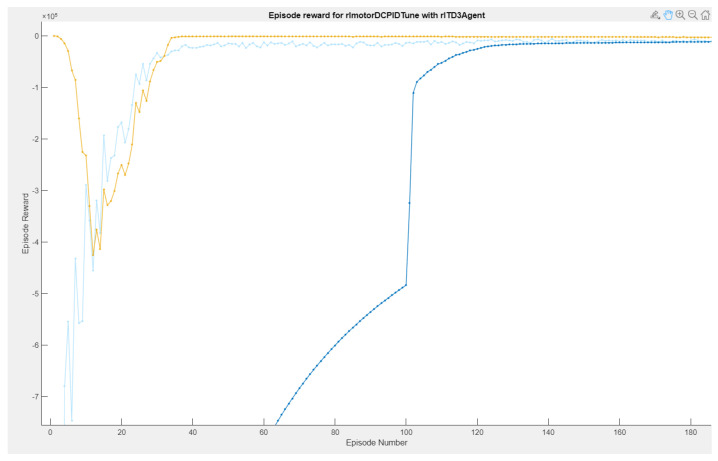
Agent RL training reward episodes.

**Figure 16:**
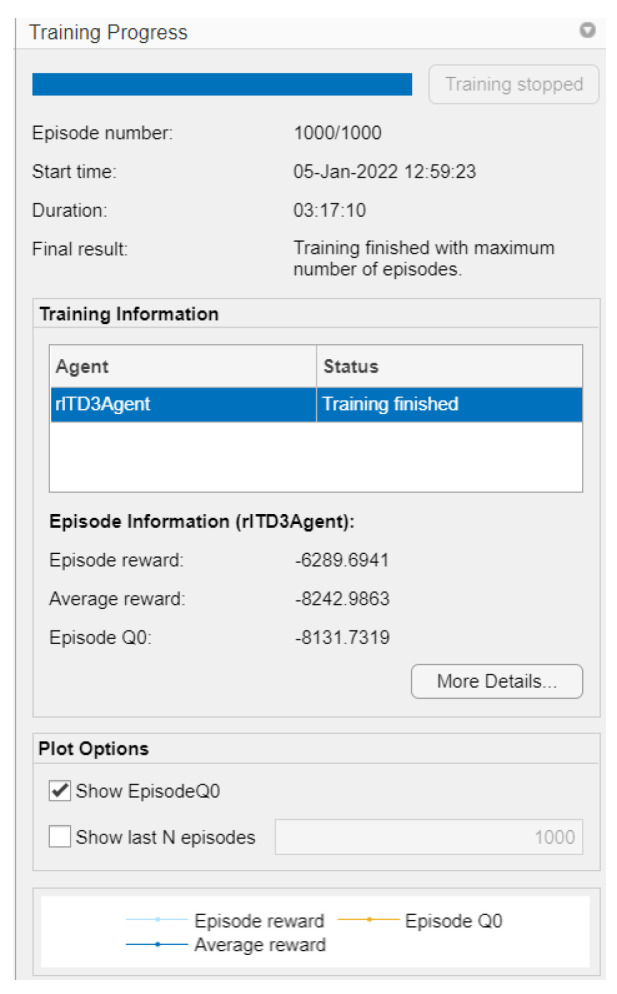
General training conditions.

While the graph of the Q0 episodes is the consecutive learning progress carried out by the agent’s actions, so that the critic learns from the system, this critic is the one that defines which are the controller gains to be adjusted in the adaptive PID control (as shown in Figure 15. In Figure 16, it can be observed that the training time was 3 h:17 m:10 s with 1000 simulation episodes, where it is considered that the agent is fully trained and the weights of the critics are the values of the controller’s gains.

### 4.2. Training of the NARMA-L2 Controller

**Figure 17:**
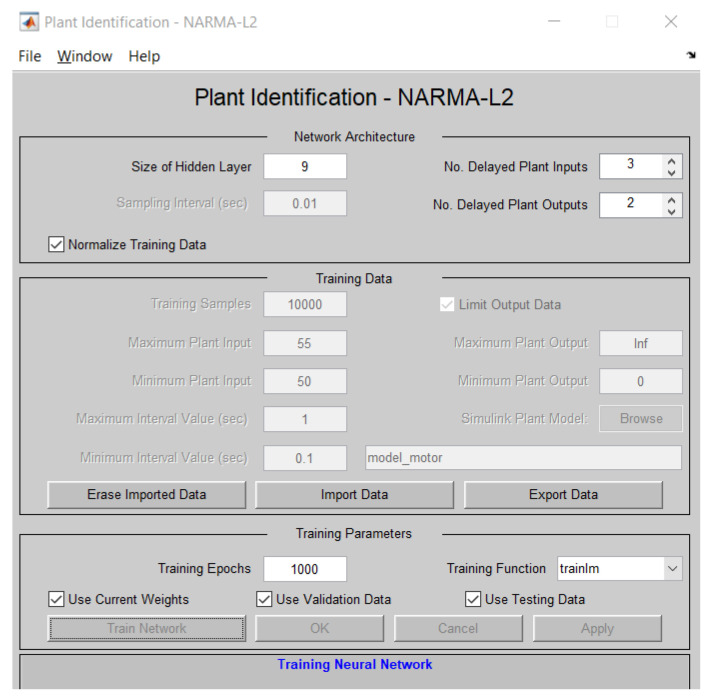
Plant identification setup.

The controller’s training was carried out based on the identification of the plant, which was determined by a Simulink model specifying the inputs and outputs of the system, as shown in Figure 17.

**Figure 18:**
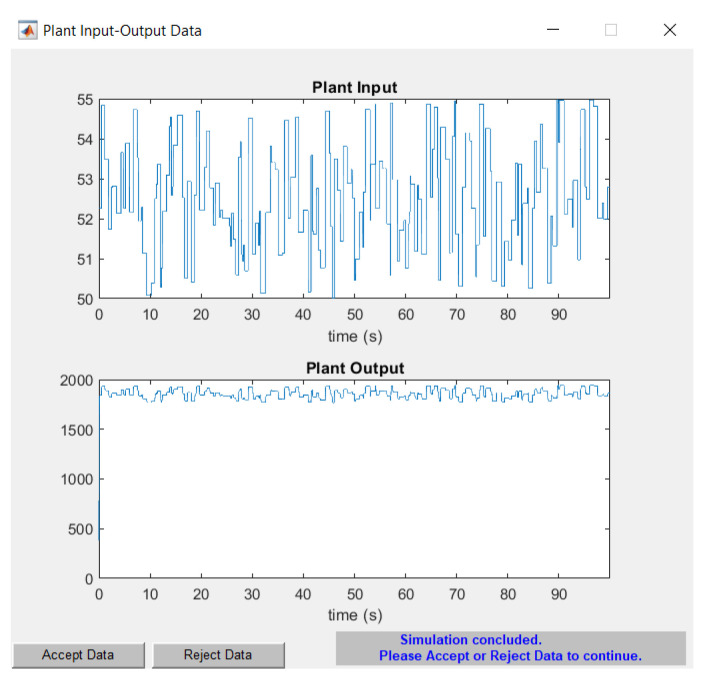
Acceptance/rejection of plant identification.

Once the plant is identified by the NARMA-L2 controller, the neural network is trained. For this purpose, the data are rejected or accepted based on the results obtained in Figure 18. If the data are rejected, adjustments must be made to the training data. The output oscillates around the reference value of 1800 RPM, which is crucial for determining the error when testing the controller.

**Figure 19:**
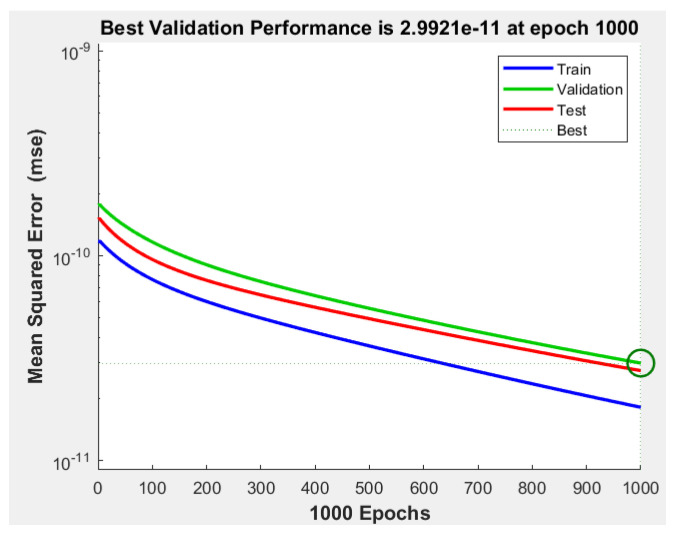
MSE for training, validation, and testing.

Similar training characteristics to those indicated in the reinforcement learning model are applied, involving 1000 episodes to train the NARMA-L2 controller. To assess its error, the Mean Squared Error (MSE) curves shown in Figure 19 are provided, indicating an approximate validation performance of $2.9921\times 10^{-11}$ over the 1000 episodes.

### 4.3. Controller Efficiency

The gains of the PI controllers obtained by the classical model and through reinforcement learning training are shown in Table 4.

**Figure 20:**
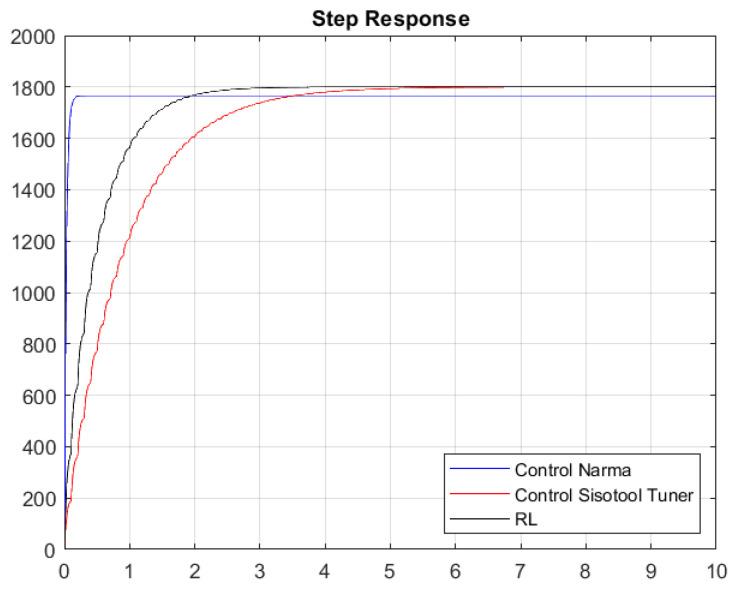
Step response without considering white noise.

**Figure 21:**
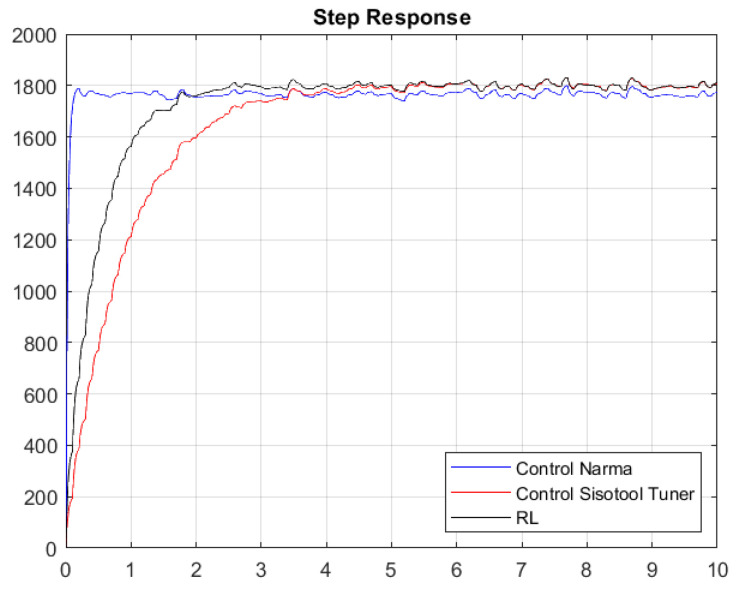
Step response considering white noise.

Once the gains of the controllers are obtained with the first Simulink model, the system response shown in Figure 20 and Figure 21 is obtained.

Figure 20 and Figure 21 present the results of the constants adjusted by the user and those obtained by means of the RL algorithm and the NARMA-L2 controller. The system contains white noise; for this reason, there are small variations during its journey in the stabilization process, which is the reference point of 1800 RPM. Graphically, it can be seen that the system has an overdamped behavior and that the RL algorithm has a better response than the one set by the user, because it has a shorter stabilization time and, therefore, the damping constant is lower. The responses in steady state are very similar when comparing the RL Agent to classical control, and both reach their target, which is the desired reference point. Conversely, if we compare the RL Agent with the NARMA-L2 controller, there is a difference in the reference point, where the RL Agent approaches the one adjusted by the user.

Table 5 and Table 6 specify the characteristics of the system obtained with the closed-loop simulation of the PI constants, as well as those obtained by the Sisotool and the RL algorithm; similarly, the metrics of the step response with respect to the NARMA-L2 controller are provided. As indicated graphically, the RL algorithm presents a better performance in the obtained response compared with the other two controllers. The RL agent achieves a faster settling time than the classical controller, while its reference value has an error of 0% compared with the NARMA-L2 controller, which has an error of 2%.

Without considering the noise, we can observe that its steady-state value tends to reach the speed set by the user. It is true that the time in steady state is better with the RL algorithm, thus having better variations between the action signal, while their percentage over level is very similar. The NARMA-L2 controller exhibits a better settling time, but its steady-state value does not align with the specified 1800 RPM.

With response to white noise, a margin of around 3.0315 s of difference was obtained in a comparison between the classical control and the RL agent, which is similar to the peak time, in which there is a margin of 0.9386 s. However, the disturbances due to the incorporated white noise cause the system to present certain peaks that lead to a percentage over level, which is 1.1825% lower in the optimal algorithm. Finally, the maximum values of the peaks in the system turn out to be equal. Regarding the comparison between the NARMA-L2 controller and the RL Agent, these two controls exhibit very favorable characteristics compared with the classical controller. They were defined based on neural network training. However, the RL Agent achieves a higher approximation to the reference value than the NARMA-L2 controller, as the latter oscillates at values below 1800 RPM. After hours of training, it can be indicated that reinforcement learning allows us to achieve similar (and even superior) control compared with using classical techniques, such as the Sisotool in MATLAB. When experiencing significant changes or disturbances in the system, the outcome of the classical PI controller might vary. Therefore, by providing an alternative that enables us to maintain system reliability in terms of robustness and stability, reinforcement learning stands as a reliable tool for use in automatic controls.

Table 7 indicates that both controllers produce stable responses, while the controller adjusted by Sisotool produces a slower response. However, the RL tuning method produces a higher gain margin and optimal solution with shorter time, thus having a much faster desired speed. The gains found by the RL algorithm take less effort compared with the gains adjusted in Sisotool. We can see this in the cost values that are closer to zero than with the one proposed by the user, both with or without white noise. In order to enhance understanding, promote transparency, and provide a guide on how to replicate the current work, the following link to the GitHub repository (https://github.com/ualejand/Reinforcement_Learning) is included. This resource enables the scientific community to examine and reproduce our work, thereby facilitating a deeper understanding of the methodology and the results obtained.

## 5. Conclusions and Future Works

The conclusions are presented in relation to the (i) policy used in the algorithm, (ii) the analysis of the LQG cost function, (iii) the comparison of the use of control techniques, and (iv) the computational capabilities in the development of learning. (i) MATLAB 2021b software was used to implement a speed control algorithm for a DC motor, which allowed for obtaining adaptive PI constants based on reinforcement learning. The policy used was the DDPG TD3 type, which contained the actor and critic formed by neural networks capable of establishing the RL agent. The networks were driven by 300 neurons that constantly learned from rewards and evaluated how optimal the control signal was using the LQG criterion cost function, indicating the least effort of the controller to reach the reference signal. Two critics allowed obtaining the weights of the proportional and integral controllers, respectively, after the RL agent was trained.

(ii) The PI controller generated by the reinforcement learning algorithm achieved higher performance than the controller designed by NARMA-L2 y Sisotool; the latter one affects the classical control. The step response characteristic of the RL algorithm exhibited an overdamped response, with a shorter settling time and steady-state error close to zero. The LQG cost function indicated that the RL controller executes a control signal with the least effort to reach its reference point. With or without white noise, there is a difference of around $6.7392\times 10^{6}$ between the cost functions, highlighting that the adaptive PI control based on RL exhibits higher efficiency. The NARMA-L2 controller exhibits a fast settling time, but its reference value has a higher error, because it does not reach 1800 RPM. The RL agent provides a closer approximation, and this trend is observed in both noisy and Gaussian-noise-free data. (iii) In the present study, two control techniques were addressed: a classical approach and a modern one involving artificial intelligence concepts. With the classical technique used by MATLAB’s Sisotool, it is evident that robustness and stability depend on the designer’s choices and conditions. In contrast, the reinforcement learning technique relies on neural networks, the number of episodes, and computational resources, among other factors. However, the engineering process of obtaining a transfer function (mathematical system modeling) often demands in-depth physical concepts and is contingent on the materials used in the control plant’s design. If some initial conditions change (such as temperature or maintenance), the engineering process must be repeated to obtain an appropriate controller. Conversely, with the DDPG TD3 training policy, the system continually adjusts itself, eliminating the need for the user to engage in re-engineering for applied control.

(iv) It is important to highlight that the better the computational resources where the algorithm is developed, the less time it will take to obtain the gains of the PI controller. In this case, the development took approximately 3 h, which allowed us to obtain a similar adjustment to that obtained by the classical control. For the NARMA controller, to adjust the plant to the reference value, its identification was carried out first, followed by its training, which took approximately 3 h. Neural networks contribute 70% of the computational costs due to the required mathematical processing, which constantly iterates depending on the average gain resulting from the algorithm.

As future work, we intend to use the algorithm on open-source hardware, such as a Raspberry PI, using Python code so that anyone can control the system through reinforcement learning. We will analyze LQG costs and consider the necessary neural networks to find the gains of the PI controller. In the event that the real control system undergoes changes in its conditions, it is possible to maintain the stability of the plant. However, this might lead to studies on predictive maintenance in order to ensure efforts to uphold the system’s robustness and stability.

## Figures and Tables

**Table 1:** DC motor system constants.

Symbol	Description	Unit
*J*	Rotor moment of inertia	kg·m^2^
*B*	Viscous friction constant of the motor	N·m·s
*R*	Electric resistance	Ω
*L*	Electrical inductance	H
*ka*	Electromotive force constant	$\frac{V\cdot s}{\mathrm{rad}}$
*km*	Motor torque constant	$\frac{N\cdot m}{A}$

**Table 2:** Characteristics of the DC motor.

Model	TH45S
Make	TYHE
Supply voltage	12 [Vdc]
Torque	100 [N m]
Speed range	2000–6000 [RPM]
Nominal current	3.5 [A]
Efficiency	IE2

**Table 3:** Technical specifications of the computer.

Operative System	Windows 10 Pro-64 bits
Processor	AMD Ryzen 7 3700U
Processor cores	8
Processor frequency	2.3 GHz
Graphics card	Radeon Vega Mobile Gfx
VRam memory	2034 Mb
Ram memory	16384 Mb
Hard disk	256 GB Type SSD + 1 TB Type HDD

**Table 4:** Gain of PI controllers.

Type Gain	Control Sisotool Tuner	RL Agent
*Proportional constant*	0.0031	0.0061
*Integral constant*	0.0299	0.0525

**Table 5:** System response without considering Gaussian noise.

Type of Controller	Peak Time	Transient Time	Steady State Time	Percentage over Level	Peak Value
*Control Sisotool Tuner*	1.9552	3.5020	3.5020	0.0016	$1.8000\times 10^{3}$
*RL Agent*	1.0973	1.9246	1.9246	0.0017	$1.8000\times 10^{3}$
*NARMA-L2 Controller*	0.0624	0.1169	0.1169	0.0004	$1.766\times 10^{3}$

**Table 6:** System response considering Gaussian noise.

Type of Controller	Peak Time	Transient Time	Steady State Time	Percentage over Level	Peak Value
*Control Sisotool Tuner*	2.0450	5.2015	5.2015	1.2945	$1.8314\times 10^{3}$
*RL Agent*	1.1064	2.1700	2.1700	1.1825	$1.8310\times 10^{3}$
*NARMA-L2 Controller*	0.0628	0.1135	0.1135	0.0004	$1.7992\times 10^{3}$

**Table 7:** Costs determined by LQG criteria.

Costs	Peak Time	Transient Time
*Sisotool Tuner*	−$1.6170\times 10^{7}$	−$1.6201\times 10^{7}$
*Reinforcement learning*	−$9.4182\times 10^{6}$	−$9.4434\times 10^{6}$

## Data Availability

In order to ensure the reproducibility of our work, we have included the GitHub repository (https://github.com/ualejand/Reinforcement_Learning), containing all the data and methods developed.
